# Affinity-Based Magnetic Nanoparticle Development for Cancer Stem Cell Isolation

**DOI:** 10.3390/polym16020196

**Published:** 2024-01-09

**Authors:** Cansu İlke Kuru, Fulden Ulucan-Karnak, Büşra Dayıoğlu, Mert Şahinler, Aylin Şendemir, Sinan Akgöl

**Affiliations:** 1 Department of Biochemistry, Faculty of Science, Ege University, 35100 İzmir, Turkey; cansuilke89@gmail.com (C.İ.K.); sinan.akgol@ege.edu.tr (S.A.); 2 Department of Bioengineering, Faculty of Engineering, Ege University, 35100 İzmir, Turkey; bsrdayioglu@gmail.com (B.D.); mertsahinler@hotmail.com (M.Ş.); aylin.sendemir@ege.edu.tr (A.Ş.)

**Keywords:** magnetic nanoparticle, cancer stem cell, affinity interactions, stem cell isolation

## Abstract

Cancer is still the leading cause of death in the world despite the developing research and treatment opportunities. Failure of these treatments is generally associated with cancer stem cells (CSCs), which cause metastasis and are defined by their resistance to radio- and chemotherapy. Although known stem cell isolation methods are not sufficient for CSC isolation, they also bring a burden in terms of cost. The aim of this study is to develop a high-efficiency, low-cost, specific method for cancer stem cell isolation with magnetic functional nanoparticles. This study, unlike the stem cell isolation techniques (MACS, FACS) used today, was aimed to isolate cancer stem cells (separation of CD133^+^ cells) with nanoparticles with specific affinity and modification properties. For this purpose, affinity-based magnetic nanoparticles were synthesized and characterized by providing surface activity and chemical reactivity, as well as making surface modifications necessary for both lectin affinity and metal affinity interactions. In the other part of the study, synthesized and characterized functional polymeric magnetic nanoparticles were used for the isolation of CSC from the human osteosarcoma cancer cell line (SAOS-2) with a cancer stem cell subpopulation bearing the CD133 surface marker. The success and efficiency of separation after stem cell isolation were evaluated via the MACS and FACS methods. As a result, when the His-graft-mg-p(HEMA) nanoparticle was used at a concentration of 0.1 µg/mL for 10^6^ and 10^8^ cells, superior separation efficiency to commercial microbeads was obtained.

## 1. Introduction

Stem cells are defined as cells that can self-renew and transform into specific cells and are used in many clinical and therapeutic applications. Stem cells exist both in embryos and adult cells [1,2]. Stem cells can form new tissue and repair damaged tissues with their self-renewal and differentiation properties [3,4].

Stem cells cover an important area in terms of stem cell applications in skin tissue engineering, and they are defined as a potential treatment tool in the treatment of neural diseases such as Parkinson’s and Alzheimer’s [1,5,6,7,8]. However, the use of stem cells is not limited to regenerative medicine. Recently, it has been determined that a group of tumor cells showing stem cell characteristics plays an important role in cancer formation and metastasis. The development of new therapeutic agents is aimed at overcoming the radiotherapy and chemotherapy resistance of this subgroup called cancer stem cells [9,10,11].

Cancer stem cells (CSCs) are a tiny subpopulation of tumor cells that can self-renew, differentiate, and become tumor-producing when injected into an animal host [12,13,14]. This cell group has been named “cancer stem cells” (CSC) because they contain various stemness genes, have an important regeneration capacity, and can differentiate into different phenotypes. Since it has not been clarified yet, the terms “tumor-initiating cell” or “tumor-initiating stem-like cell” have been suggested instead of the term “cancer stem cell”, since they initiate tumor development, which is one of the defining characteristics of these cells [15,16]. The inadequacy of cancer treatments has been associated with cancer stem cells that cause metastasis and are resistant to radio and chemotherapy. Systematic examination of the genetics, biological properties, and signal transduction mechanisms of cancer stem cells will help to elucidate the mechanisms of carcinogenesis. Although chemo/radiotherapy destroys most cells in the tumor, cancer stem cells may remain. These cells may form tumors again due to increased resistance. The development of effective and reliable treatment methods targeting cancer stem cells depends on the isolation and identification of these cells from cancer cell lines and tumor tissues [17,18].

To improve industrial applications for drug target discovery and predictive toxicity, and to acquire a better understanding of their potential for tissue regeneration, stem cells are crucial tools. They also help to further our understanding of general cellular processes [19]. Therapeutic cell separation is used in medicine to introduce enriched cell populations to patients who have a medical need for them. Examples of this include enriching hematopoietic stem cells using immunomagnetic separation or separating leukocytes via aphaeresis. Additionally, it makes it possible to count the cells in a person’s blood system, and it can help patients with multiple sclerosis who have received immunoablation therapy to repopulate their immune systems [20].

Among the stem cell isolation methods, the most preferred methods are cell surface marker-based separation methods [21]. Studies are limited due to the high cost/efficiency ratios of surface marker-based separation methods [22]. For this purpose, innovative stem cell isolation methods with a low cost/efficiency ratio are needed in order to develop CSC studies. Although the most widely used isolation methods are known as magnetically labeled stem cell separation (MACS) and fluorescently labeled stem cell separation (FACS), dielectrophoresis (DEP) and density difference stem cell separation methods are also used [19,23]. However, methods other than MACS and FACS are not preferred because stem cells are generally characterized by surface markers [24]. In addition to the advantages of these two separation methods, which are popularly used today, there are disadvantages in terms of cost, efficiency, and time. Due to the high cost of FACS separation, small- and medium-sized research groups purchase FACS services within their budgets. However, due to the slow system of the FACS system, the performing times are becoming longer; therefore, researchers are turning to the MACS system. However, the fact that MACS separation can only be used in a certain cell number range and has a low-efficiency rate affects the efficiency of the studies [25].

Today, although many CD markers are stem cell markers, they are also used to identify cancer stem cells. Although not all cancer stem cell markers mentioned in some studies are found in all cancer types, some cells found in small amounts in tumors have been shown to support tumor formation and contain many stem cell markers in the literature. CD133 stem cell floating antigen, also known as promin or PROML1, has been simultaneously shown as a CHD marker in brain, colon, and prostate cancers [26,27]. The marker identified in cancer stem cells of brain tumors such as glioblastoma and neuroblastoma is CD133^+^. When CD133-positive cells and CD133-negative cells were isolated and cultured in different dishes under the same conditions, CD133-positive tumor cells formed tumor spheres and the cell number increased, whereas CD133-negative cells did not proliferate and did not form tumor spheres [28]. Likewise, CD133 expression was analyzed in osteosarcoma and SaOS-2 cells by cell culture and quantitative PCR techniques [29]. In another study, it was determined that cancer stem cells isolated according to the CD133 marker are rich in GalNAc (α-N-acetylgalactosamine). Accordingly, it has been shown that cancer stem cells selected by the GalNAc-recognizing lectin *Dolichos biflorus agglutinin* (DBA) show similar characteristics to cells differentiated according to the CD133^+^ cell marker and form a cell in vivo [30]. In this respect, DBA can be used as a specific recognizer for CD133^+^ cells, and many studies have shown in the literature that it has diagnostic properties for stem cells [31,32].

Due to the distinctive structural and functional characteristics of nanomaterials, nanotechnology can address the issues of insufficient capture efficiency and low purity of CTCs, indicating a high potential for CTC separation and detection. Considering the metastasis characteristics of cancer stem cells and the possibility and level of metastasis achieved by isolating cancer stem cells from patients, it is planned that the developed magnetic nanoparticles will outperform the magnetic nanoparticles used in the literature and that an efficient isolation method will be developed by applying them to microfluidic systems in the future [33,34].

The main purpose of this study is to develop a new stem cell isolation method using affinity-based magnetic polymeric nanoparticles. Surface activity, chemical reactivity, and surface modifications required for both lectin affinity and metal affinity interactions were added to the produced and described affinity-based magnetic nanoparticles. Following sterilization, cytotoxicity tests, and the separation of CSC from the human osteosarcoma cancer cell line (SAOS-2) with a cancer stem cell subpopulation harboring the CD133 surface marker, the functional polymeric magnetic nanoparticles that had been produced and described were employed.

## 2. Method

### 2.1. Synthesis of Magnetic p(HEMA) Nanoparticles

The mini-emulsion method was used for the synthesis of magnetic p(HEMA) nanoparticles. For this purpose, Phase 1 (aqueous phase) was prepared by dissolving 0.4 g of PVA 57.7 mg of SDS and 46.9 mg of NaHCO_3_ in 200 mL of distilled water. To this phase, Phase 2 oil phase (monomer phase)—0.8 mL HEMA and 4.2 mL EGDMA—was added. The prepared mixture was mixed in a homogenizer at 800 rpm for 15 min. After homogenization, the mixture was passed through nitrogen gas for 2–3 min. In the 3rd phase, 0.25 g Fe_3_O_4_ (synthesized in the previous step), 0.2 g NaHSO_3_, and 0.3 g APS were added to the other phases and the volume was completed to 250 mL. The polymerization solution was taken to the reactor where polymerization would take place and left to stir for 24 h at 500 rpm at 40 °C. At the end of the period, ethanol–water washing was performed 3 times. Within the scope of washing, nanoparticles were centrifuged at 50,000× *g* for 20 min, and ethanol/water was added and sonicated. At the end of the washing, the nanoparticles were made ready for use [35].

### 2.2. Modification of Magnetic p(HEMA)Nanoparticles by Grafting

After the magnetic p(HEMA) nanoparticles were synthesized as previously described, His grafting was performed. The following procedures were applied to synthesize L-histidine grafted mg-p(HEMA) (His graft-mg-p(HEMA) np). A total of 1.4 g of NaH and 50 cm^3^ of tetrahydrofuran were dissolved in 20 g of p(HEMA) as a catalyst and added to the mixture containing 3 g of L-His. The grafting reaction was achieved by mixing this mixture at 40 °C for 24 h. At the end of this period, His-graft-mg-p(HEMA) was removed from the medium, and the mixture was washed several times with methanol and water to remove L-His that did not bind to p(HEMA). Obtained His graft-mg-p(HEMA) nanoparticles were stored at +4 °C for later use [36].

### 2.3. Binding of Cu^2+^ and DBA Lectin to Magnetic His-graft-p(HEMA)Nanoparticles

#### 2.3.1. Binding of Cu^2+^ to His-graft-mg-p(HEMA) Nanoparticles

For binding Cu^2+^ to His-graft-mg-p(HEMA) nanoparticles, 1000 ppm Cu (NO_3_)_2_ (copper nitrate) solution was prepared in 50 mL water adjusted to pH 6 to bind the copper atom. A total of 100 mg of His-graft-mg-p(HEMA) nanoparticles were added to this solution and mixed at room temperature for 2 h [37,38]. At the end of the time, the nanoparticles were precipitated via centrifugation at 14,500× *g* for 20 min. The amount of bound and unbound copper in the supernatant was determined spectrophotometrically using the PAR (2-pyridyl azo resorcinol) method. Within the scope of the PAR method, 0.1000 g PAR (sodium salt) was weighed and dissolved with distilled water, taken into a flask, and the volume was made up to 100 mL with bidistilled water (1000 ppm). We added 100 µL of sample solution, 200 µL of PAR reagent, 200 µL of 0.1 M pH = 9.2 sodium borate buffer, and 500 µL of d to the reaction medium. We added water and left for 10 min. Then, spectroscopic measurements were made at a wavelength of 510 nm. The solution without a sample was prepared as a blank solution [39,40]. Whether copper is added to the structure or not can be determined via the PAR method [41].

#### 2.3.2. Binding of Lectin (DBA) to His graft-mg-p(HEMA)-Cu^2+^ Nanoparticles

To bind lectin (DBA), 500 µL of DBA solution at 0.25 mg/mL concentration was added to 100 µL of His graft-mg-p(HEMA)-Cu^2+^. The solution was mixed in phosphate buffer (pH 7.4) at room temperature for 1 h. At the end of the time, the nanoparticles were precipitated via centrifugation at 14,500× *g* for 20 min. The amount of bound and unbound DBA in the upper phase was determined spectrophotometrically at 280 nm [42,43,44].

## 3. Characterization of Synthesized Magnetic Nanoparticles

Dry mass analysis, FTIR (Fourier transform infrared spectrophotometer) measurements, SEM (scanning electron microscopy) measurements, AFM (atomic force microscope) measurements, elemental analysis measurements, magnetism analysis, and zeta dimension analysis were performed within the scope of characterization of synthesized magnetic nanoparticles.

### 3.1. Dry Mass Analysis

For the dry particle mass of the synthesized nanoparticles, 0.1–0.25–0.5–0.75–1 mL of nanoparticles were added to the tared Eppendorf tubes, and the supernatants were obtained by centrifuging at 14,500× *g* for 20 min. The particles were dried by placing Eppendorf tubes in an oven at 45 °C. The dry particle mass was determined by subtracting the tare of the Eppendorf tubes from the final mass. The number of g particles per unit volume (mL) was calculated by plotting the mass-volume graph.

### 3.2. FTIR (Fourier Transform Infrared Spectrophotometer) Measurements

The FTIR spectra of the synthesized nanoparticles were obtained using the FTIR spectrophotometer (FTIR 8000 Series, Shimadzu, Kyoto, Japan). For this, dried nanoparticles (2 mg) were homogeneously mixed with KBr (98 mg, IR Grade, Merck, Darmstadt, Germany) and made into tablets, and the FTIR spectrum was drawn in the wavenumber range of 4000–400 cm^−1^ [45].

### 3.3. SEM Characterizations

To make SEM measurements, polymeric nanoparticles were dispersed on the lamella surface and dried. SEM measurements of the particle were performed using a scanning electron microscope (Thermo Scientific Apreo S, Waltham, MA, USA).

### 3.4. AFM (Atomic Force Microscope) Measurements

To obtain information about the morphology of the synthesized nanoparticles, AFM measurements were performed using atomic force microscope (BRUKER Dimension Edge, Billerica, MA, USA) with a ScanAsyst. For this, nanoparticles were dispersed on the glass surface in 1 cm × 1 cm dimensions and dried. AFM measurements were made using the tapping mode.

### 3.5. Elemental Analysis Measurements

Elemental analysis was carried out to determine whether the groups we want to be added to the synthesized nanoparticles after modifications are added and to determine their chemical composition with the EDS instrument (Thermo Scientific Apreo S, Waltham, MA, USA).

### 3.6. Magnetism Analysis

The magnetism degree of the synthesized magnetic nanoparticles and the presence of polymeric ferrite magnetite’s were determined via electron spin resonance (ESR) spectrometry [37]. Electron spin resonance (ESR) spectra of the investigated sample were transferred into sealed quartz tubes of ~4 mm internal diameter and were recorded using a ESR instrument (Bruker EMX 113 X-Band ESR, Billerica, MA, USA) spectrometer equipped with a cylindrical cavity (ER 4119HS) resonator containing a standard sample (DPPH). Signal intensities were calculated from the first derivative spectra of the absorption peak plotted against the magnetic field and compared with that obtained for the standard sample under the same spectrometer operating conditions. The measurements were made at room temperature. The spectrometer operating conditions adopted during the experiments are given in Table 1.

### 3.7. Zeta Size and Potential Analysis

Zeta size analysis of synthesized nanoparticles was analyzed with Nano Zetasizer (NanoS, Malvern Instruments, London, UK). For this purpose, particle zeta size measurement was performed by placing 1 mL of nanoparticle solution in the sample chamber of the zeta size analysis device [46].

## 4. Sterilization of Nanoparticles

Sterilization of nanoparticles used in cell culture studies was achieved with 70% ethanol. Thus, it was aimed to reduce the agglomeration, which is a characteristic feature of nanoparticles, during the drying and redistribution process. Ethanol sterilization of His-graft-mg-p(HEMA) nanoparticles was carried out primarily, considering the possibility that DBA, which is the last stage of nanoparticle modification, is in the protein structure on the surface, in its DBA-bound state, to be negatively affected by ethanol. All modification steps were then performed in sterile environments. Briefly, His-graft-mg-p(HEMA) nanoparticles were incubated in 70% ethanol with gentle stirring for 24 h. Then, nanoparticles were washed several times with sterile ultrapure water to remove ethanol. For Cu^2+^ modification, the Cu^2+^ solution prepared using sterile distilled water was filtered through a 0.2 µm sterile syringe filter and the modification was performed as described above. The solution used for DBA modification was also prepared in sterile phosphate buffer and binding was performed as above. All these stages were completed in a sterile cabinet. Nanoparticles were freshly prepared before each application. The materials prepared with a nanoparticle concentration of 1 mg/mL were stored at +4 °C to be used as stock in the experiments.

For the sterilization of nanoparticles, the final product, His-graft-mg-p(HEMA)-Cu^2+^-DBA nanoparticles, removes tryptic soy broth (TSB, Sigma-Aldrich, St. Louis, MO, USA) and thioglutalate broth (TGB, Sigma-Aldrich, St. Louis, MO, USA) contamination media. It was carried out by inoculating in tubes containing water and incubating at 37 °C for 7 days and observing the turbidity of the media [47].

Tryptic soy broth and thioglutalate broth were prepared by dissolving 0.03 g/mL tryptic soy and thioglutalate in ultrapure water following the manufacturer’s instructions and sterilized by autoclaving at 121 °C for 45 min and aliquoted into 9 mL contamination tubes under a laminar flow cabinet.

## 5. Cell Culture

In this study, SAOS-2 (SAoS-2/An1 (Human), Human bone osteosarcoma from HÜKÜK, Republic of Turkey, Ministry of Agriculture and Forestry, Institute of Foot and Mouth Disease, Ankara, Turkey, Accession number: 02111901) and HEK-293 (ATCC 293T with an accession number of crl-3216) cell lines were used. Cells were grown in vitro following cell culture techniques at 37 °C in 95% humidity containing 5% CO_2_. SAOS-2 and HEK-293 cell lines, DMEM F12 (Merck, Darmstadt, Germany) medium, 10% (*v*/*v*) inactive fetal bovine serum (FBS; Biochrom, Berlin, Germany), 1% (*v*/*v*) L-glutamine (Biochrom, Germany) and 1% Penicillin-Streptomycin.

### 5.1. Preparation of the Cells

The cells in the cryotube were removed from the liquid nitrogen tank or the deep freezer at −86 °C and quickly thawed in a water bath set at 37 °C. The mouth of the cryotube was loosened, 1 mL of ready-made nutrient medium containing serum was added to the cells drop by drop, and the cells were collected with a pipette and then transferred to the centrifuge tube. The medium was added to the tubes so that the total volume was 10 mL. Then, it was centrifuged 1000 rpm at 4 °C for 5 min. By discarding the supernatant formed after centrifugation, DMSO in the freezing medium (90% (*v*/*v*) FBS, 10% (*v*/*v*) DMSO) was removed, the pellet remaining at the bottom was homogenized with the nutrient medium and transferred to appropriate culture vessels (175 cm^2^). Cells were incubated at 37 °C in a 95% humidity environment containing 5% CO_2_ for 24 h. Then morphology and adherence rates were evaluated with a reversed-phase microscope (Olympus, Tokyo, Japan). Cells were passaged before in vitro experiments, changing the medium every 2 days, when they reached 80% confluency.

### 5.2. Passaging and Stocking of Cells

The medium used by the cells in the flask was removed. Then, to remove serum and Ca^2+^-Mg^2+^ ions, PBS (phosphate buffer solution; (0.157% (*w*/*v*) Na_2_HPO_4_·2H_2_O, 0.08% (*w*/*v*) NaCl), heated at 37 °C, the cultures were washed with 0.2% (*w*/*v*) KCl, ultrapure H_2_O). A solution of trypsin-EDTA (2.5 g/L trypsin, 0.5 mM EDTA) pre-warmed at 37 °C was added so that the cell culture dishes were kept at 37 °C in a CO_2_-free incubator for 5 min. We examined whether the cells were removed from the surface. The culture dishes were mixed with serum containing 2–5 times the volume of trypsin-EDTA. The cells homogenized in the medium were taken into centrifuge tubes. Tubes were centrifuged for 5 min at 4 °C at 1000 rpm. The supernatant was poured, resuspended in a pellet medium, and transferred to culture dishes. Cells were passaged into culture dishes at a ratio of 1:2 or 1:3. Culture dishes were incubated at 37 °C in a 95% humidity environment containing 5% CO_2_.

Cells were stocked so that the study could be performed within a certain cell passage number range. For this, cells were stored in freezing tubes in freshly prepared freezing medium (90% (*v*/*v*) FBS, 10% (*v*/*v*) DMSO). The freezing tubes were raised to −86 °C or −196 °C (liquid nitrogen) depending on the required storage time of the cells.

### 5.3. Counting Cells

Cell counting was performed to obtain the number of cells needed during the experiment. A total of 50 µL of the homogenized cell suspension was taken and placed in an Eppendorf tube, and 50 µL of trypan blue was added to 400 µL of PBS. An amount of 50 µL of the cell suspension, diluted 1:10, was taken and transferred to a Neubauer hemocytometer. Cells were counted at 10× magnification under a reverse-phase light microscope. Cells in four different areas of the Neubauer slide containing 16 small squares were counted and averaged. Cell concentration was calculated according to the following equation (Equation (1)):

(1) $$Calculation\;\left(\frac{number\;of\;cells}{mL}\right)=(\frac{number\;of\;viable\;cells\;counted\times dilution\;factor\times 10^{4}}{square\;counted}).$$

### 5.4. MTT Test

Human osteosarcoma (SAOS-2) and embryonic kidney epithelial cell lines (HEK-293) were thawed with 96-well cell culture at a concentration of 1 × 10^5^ cells/mL (1 × 10^4^ cells/well). They were cultivated in four replicates in 100 µL volumes each. Cells were incubated for 24 h in a humid incubator at 37 °C containing 5% CO_2_. After 24 h of incubation, the medium was renewed with fresh medium containing NPs at five different concentrations (0.1, 1, 10, 100, 600 μg/mL). Cells were incubated with NPs for 24 h. At the end of these periods, a viability test was performed with MTT (3-(4,5-dimethylthiazol-2-yl)-2,5-diphenyltetrazolium bromide, yellow tetrazolium salt, M5655, Sigma, St. Louis, MO, USA). Before MTT analysis, morphological examinations 48 h after cell cultivation were performed under an Olympus Ix71 Inverted Research microscope and photographed by DP71 Camera Instruction.

For MTT, the used nutrient medium in the plate was withdrawn. Serum-free medium containing 10% MTT solution at a concentration of 5 mg/mL was added to the cells. Cells were incubated in the dark at 37 °C in a 5% CO_2_ incubator for 3 h. After 3 h of incubation, the medium containing MTT was withdrawn. DMSO (41646, Sigma, St. Louis, MO, USA) was added to the cells to dissolve the formed formazan crystals. It was ensured that the crystals were dissolved thoroughly by shaking in the shaker at rpm. Absorbance values were recorded by reading at 570 nm wavelength in an ELISA reader (BioTek, Elx800).

Calibration curves were created for both cell lines to determine the cell number from the absorbance values. For this, cell cultivation was carried out in four repetitions by applying half dilution from 10^5^ cells/100 µL concentration to 10^2^ cells/100 µL concentration in 96-well cell culture dishes (24 h). MTT analysis was then performed. A graph was created with the obtained absorbance values versus the cell number, and the equation of the obtained curve was used to convert the experimental data obtained as the absorbance value to the cell number. Cytotoxicity graphs were obtained according to cell number data.

## 6. Optimizing Stem Cell Isolation with Nanoparticles

### 6.1. Isolation of Cancer Stem Cells from SAOS-2 Cell Line via Magnetic Active Cell Sorting System (MACS)

The MACS technique is a magnetic cell separation technique based on cell separation in a magnetic field with biotinylated superparamagnetic microbeads with a diameter of about 100 nm developed according to a specific cell surface receptor [22].

Anti-biotin-linked CD133/1-biotin magnetic beads (CD133 MicroBead Kit-Tumor Tissue, 130-100-857, Miltenyi, Auburn, CA, USA), LS Columns (Miltenyi, Auburn, CA, USA) for the differentiation of stem cells carrying CD133^+^ protein, a cancer stem cell surface marker from the SAOS-2 cell line (130-042-401), and LD Columns (130-042-901, Miltenyi, Auburn, CA, USA) were used. PBS (pH: 7.2) containing 0.5% (g/mL) bovine serum albumin (BSA, A9647) (Sigma Aldrich, St. Louis, MI, USA) and 2 mM EDTA (Merck, Darmstadt, Germany) (without Ca^2+^ and Mg^2+^) as MACS buffer was used.

After the cells were suspended via trypsinization, the desired number of cells (10^6^–10^7^–10^8^) was suspended with MACS buffer. (The 10^5^ and 10^9^ cell numbers written in the application form were not tested. It is not feasible to obtain the 10^9^ cell number. The CD133^+^ cell number obtained after the MACS analysis with the 10^5^ cell number is expected to be 10^3^–10^4^ cells when the separation results from higher cell numbers are considered. Since more accurate results were obtained with higher cell numbers, experiments were continued with higher numbers than 10^5^ cells.) The medium was thoroughly removed via centrifugation at 1220 rpm for 10 min. Cell separations were performed on LS and LD columns in a midiMACS separator. After the supernatant was removed after centrifugation, the pellet was resuspended and homogenized thoroughly with 300 µL of MACS buffer. A total of 100 µL of CD133 Microbead solution was added and quickly homogenized by pipetting. It was incubated at +4 °C for 30 min on a shaker (Heidolph, Titramax, Burladingen, Germany) at 300 rpm. After incubation, 2 mL of MACS buffer was added to the cell suspension and centrifuged at 1220 rpm for 10 min.

MidiMACS separator was taken on the stand. The column was placed inside the separator. A centrifuge tube was placed under the column. A total of 3 mL of MACS buffer was passed through the column so as not to form foam. After centrifugation was completed, the pellet was resuspended with 500 µL of MACS buffer for 10^6^ and 10^7^ cell counts and 2.5 mL for 108 cell counts. As soon as the dripping of buffer through the column was finished, the cell suspension was fed into the column. After the dripping was finished, 3 × 3 mL of MACS buffer was passed through the column to remove the cells that did not adhere to the microbead. Thus, CD133^−^ cells were collected in the tube. The column was separated from the separator and placed on a new centrifuge tube. A total of 5 mL of MACS buffer was added to the column. Microbead-bound CD133^+^ cells in the column were transferred into the tube by passing the buffer through the column with the help of a plunger. The sorted CD133^+^ and CD133^−^ cells were counted in a hemocytometer.

### 6.2. Analysis of CD133^+^ Cells Sorted via MACS Technique from SAOS-2 Cell Line and Fluorescent Activated Cell Sorting (FACS) Method

CD133^+^ cells sorted via the MACS technique were centrifuged at 300× *g* for 10 min. The pellet was resuspended with 80 µL of the buffer. It was homogenized with 20 µL of FCR blocking agent (CD133 MicroBead Kit—Tumor Tissue, Miltenyi, Auburn, CA, USA 130-100-857) and then with 10 µL of Labeling Check Reagent (Miltenyi, Auburn, CA, USA 130-098-866). It was incubated for 30 min in the dark at +4 °C, shaker 300 rpm. After adding 2 mL of buffer, it was centrifuged at 300× *g* for 10 min. The pellet was resuspended with 300–500 µL of PBS and prepared for FACS analysis in Eppendorf tubes. In the FACS analysis, we tried to include the entire cell population in the gate (Gate1) as much as possible without including debris and cells close to the debris in the FSC-A/SSC-A graph. The region of CD133^+^ cells (Gate2) in the SSC-A/CD133 graph was determined according to the region that peaked in the Count/CD133 graph. As a result, the number of cells stained with the CD133^+^ marker in the whole cell population is indicated in the statistical table as %.

### 6.3. Determination of CD133^+^ Cancer Stem Cell Characterization via Fluorescence Marker (FACS) Analysis

CD133^+^ cells sorted via the MACS technique were centrifuged at 300× *g* for 10 min. The pellet was resuspended with 80 µL of the buffer. It was homogenized with 20 µL of FCR blocking agent (CD133 MicroBead Kit—Tumor Tissue, Miltenyi, 130-100-857) and then with 10 µL of Labeling Check Reagent (Miltenyi, 130-098-866). It was incubated for 30 min in the dark at +4 °C, shaker 300 rpm. After adding 2 mL of buffer, it was centrifuged at 300× *g* for 10 min. The pellet was resuspended with 300–500 µL of PBS and prepared for FACS analysis in Eppendorf tubes.

In the FACS analysis, we tried to include the entire cell population in the gate (Gate1) as much as possible without including debris and cells close to the debris in the FSC-A/SSC-A graph. The region of CD133^+^ cells (Gate2) in the SSC-A/CD133 graph was determined according to the region that peaked in the Count/CD133 graph. As a result, the number of cells stained with the CD133^+^ marker in the whole cell population is indicated in the statistical table as %.

## 7. Results

### 7.1. Determination of Cu^2+^ and DBA Binding Capacities of Synthesized Nanoparticles

The binding capacities of Cu^2+^ and DBA of the synthesized magnetic nanoparticles were calculated in terms of their *Q* value. The copper binding capacities of magnetic nanoparticles are obtained by calculating the amount of mg Cu^2+^ bound per gram of polymeric material. The following equation is used for this calculation. *Q* (mg/g) is the amount of substance bound per gram polymer, *C*_0_ (mg/mL) is the initial concentration of adsorption, *C* (mg/mL) is the end concentration of adsorption, *m* (g) is the dry mass of the polymer, and *V* (mL) is the volume of the adsorption medium (Equation (2)). DBA binding capacity is calculated similarly (Equation (2)).

(2) $$Q=\left[\left(C_{0}-C\right)/m\right]\times V$$

Cu^2+^ binding capacities of His-graft-mg-p(HEMA) nanoparticles were found as 95.99 mg/g in pH 4.75 water at room temperature.

**Figure 1:**
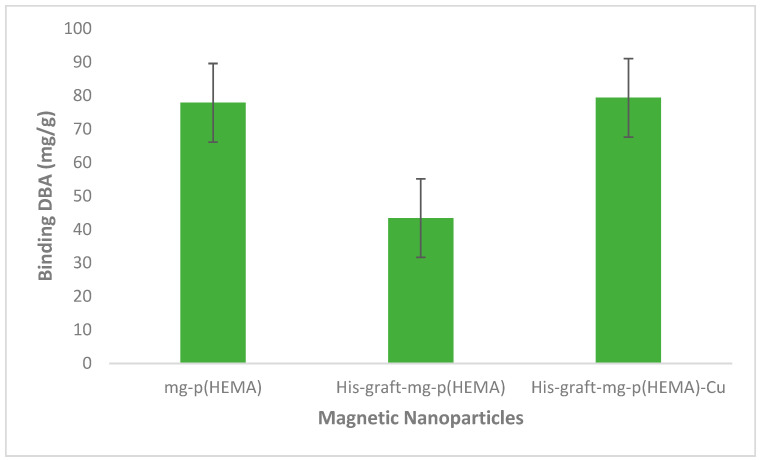
DBA binding capacities of mg-p(HEMA), His-graft-mg-p(HEMA), and His-graft-mg-p(HEMA)-Cu^2+^ magnetic particles (0.25 mg/mL DBA, pH 7.4, 0.1 M phosphate buffer, room temperature). Error bars show the %SE.

As can be seen from Figure 1, DBA binding in metal-chelated affinity systems increases slightly, and metal chelation is thought to have a stabilizing effect on the bound DBA.

### 7.2. Characterization of Synthesized Magnetic Nanoparticles

Dry mass analysis, FTIR (Fourier transform infrared spectrophotometer) measurements, SEM (scanning electron microscope) measurements, AFM (atomic force microscope) measurements, elemental analysis measurements, magnetism analysis, and zeta size analysis were performed within the scope of characterization of synthesized magnetic nanoparticles.

#### 7.2.1. Dry Mass Graph

Dry mass graphs can be found in the Supplementary file as Figure S1. These graphs were used in the calculation of Q values with Equation (2).

#### 7.2.2. FTIR (Fourier Transform Infrared Spectrophotometer) Analysis

**Figure 2:**
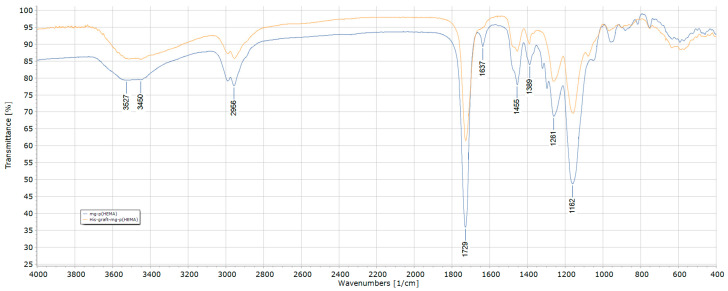
FTIR spectra of mg-p(HEMA) and His-graft-mg-p(HEMA) nanoparticles.

As seen in Figure 2, peaks around 560 cm^−1^ originating from Fe_3_O_4_ (magnetite) are seen in both spectra, and it is understood that magnetite structures participate in all nanoparticle structures.

Around 3500 cm^−1^, the –OH bands became evident, especially in nanoparticles containing p(HEMA) structures. A decrease in –OH bands is observed in histidine-bound nanoparticles. In addition, the peaks showing C=O bonds around 1729 cm^−1^ decreased due to histidine binding. There is a change in the peaks originating from C-O and C-N bonds around 1162 cm^−1^ and 1261 cm^−1^. In this case, it can be said that the amino acid histidine is included in the mg-p(HEMA) structure.

#### 7.2.3. SEM (Scanning Electron Microscope) Analysis

**Figure 3:**
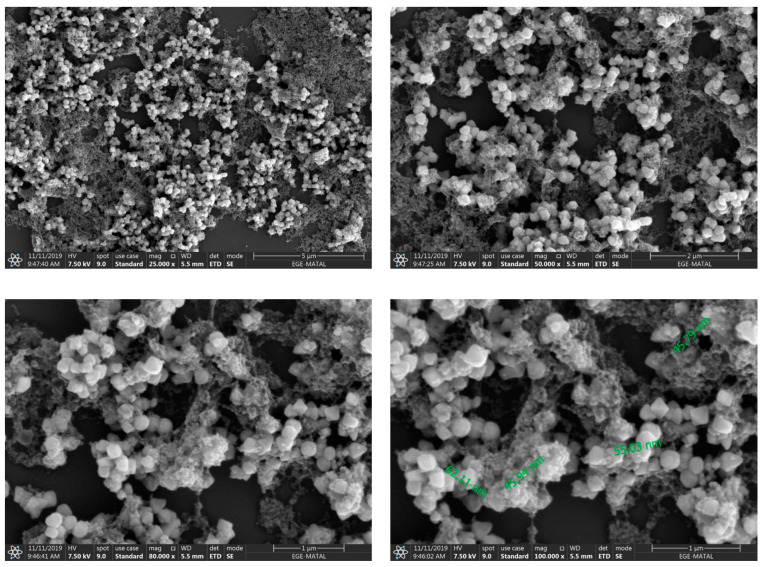
SEM images of mg-p(HEMA) np (25 kx, 50 kx, 80 kx, 100 kx).

**Figure 4:**
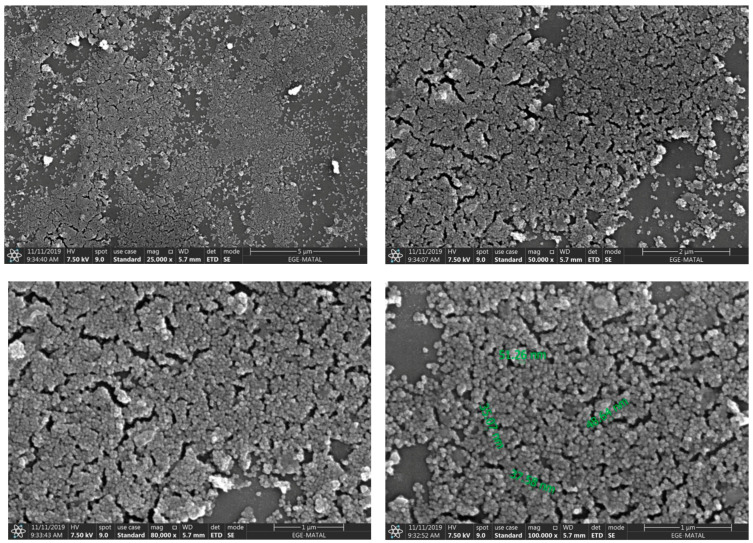
SEM images of His-graft-mg-p(HEMA) np (25 kx, 50 kx, 80 kx, 100 kx).

When the SEM images are examined, it is seen that the mg-p(HEMA) nanoparticles are in a reticulated and spherical shape, with a size of approximately 50 nm. His-graft-mg-p(HEMA) nanoparticles are approximately 45 nm and spherical in shape. It can be said that grafting with histidine does not cause a significant change in particle sizes (Figure 3 and Figure 4).

#### 7.2.4. Atomic Force Microscopy (AFM) Analysis

Ra is the arithmetic or centerline mean, Rq is the root mean square, and Rmax is the highest level of surface roughness [48]. Ra is frequently used to describe how rough modified surfaces are. It helps spot broad variations in the characteristics of the overall profile height and keep track of an established production process. Following the square of amplitude used in the calculation of Ra, the Rq value is more susceptible to higher peaks and valleys [49,50].

**Figure 5:**
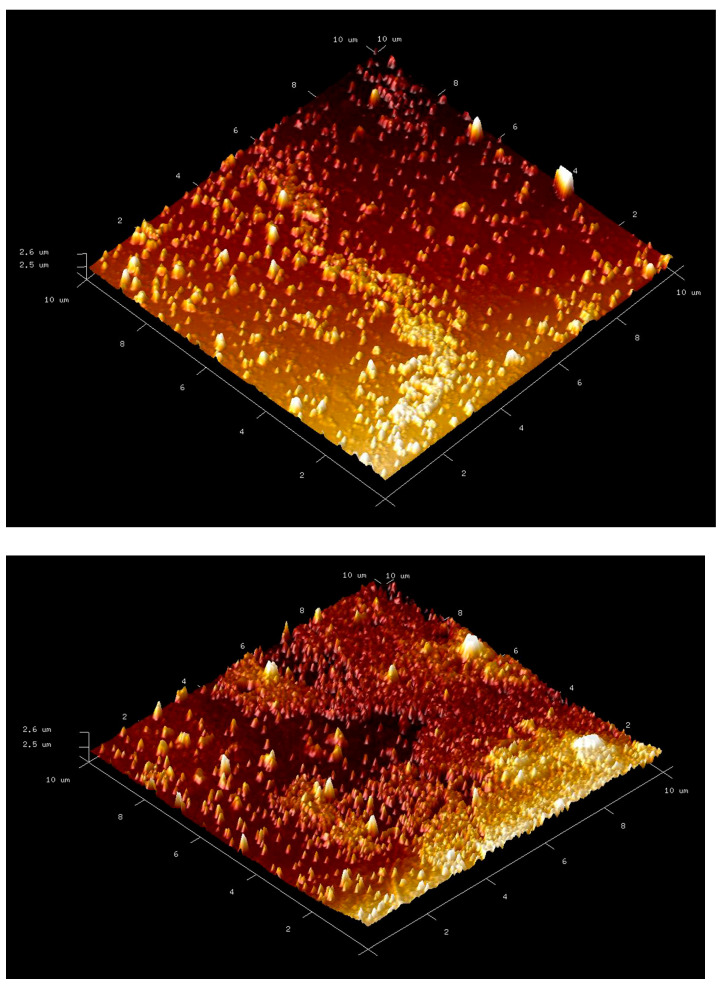
AFM image of mg-p(HEMA) and His-graft-mg-p(HEMA) np.

As can be seen in Table 2, the values of Ra and Rq are almost similar. But Rmax is decreased with the His graft procedure. It can be said that modification and derivation of mg-p(HEMA) NPs resulted in changes in surface topology and porosity (Figure 5). An increase in the surface porosity of the nanopolymer grafted with histidine is observed. In other words, it can be said that the nanopolymer surface has become rougher thanks to the surface modification.

#### 7.2.5. Energy Dispersive Spectrometry (EDS) Analysis

The presence of functional groups involved in the His-graft-mg-p(HEMA) structure after modifications to the synthesized mg-p(HEMA) nanoparticles was investigated via SEM-EDS analysis.

From the EDS analysis results shown in Table 3, it is understood from the presence of the Fe atom that Fe_3_O_4_ magnetites participate in the nanoparticle structure. With the grafting of the amino acid histidine to the mg-p(HEMA) nanoparticle, a decrease in carbon and oxygen in terms of atomic percentages was observed, while an increase in nitrogen was observed. With this increase, it can be interpreted that histidine has been added to the structure.

#### 7.2.6. Electron Spin Resonance (ESR) Analysis

**Figure 6:**
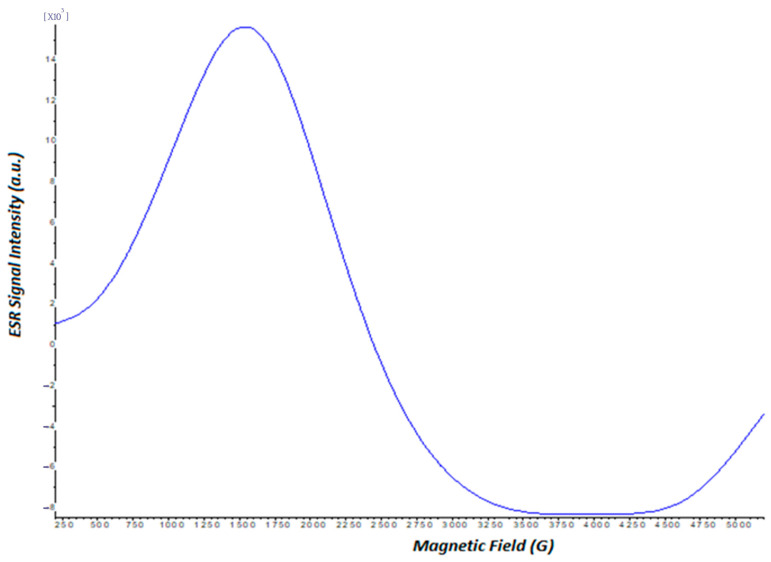
ESR spectrum for mg-p(HEMA).

The magnetism degree of the synthesized magnetic nanoparticles and the presence of polymeric ferrite magnetites were determined via electron spin resonance (ESR) spectrometry (Bruker EMX 113 X-Band ESR, Billerica, Massachusetts, USA). The presence of magnetic units included in the investigated sample was recorded via the ESR technique in Figure 6.

(3) $$g=\;\mathrm{hv}/\left(\mathrm{BHr}\right)$$

Equation (3) is used to calculate the g factor, which is a characteristic numerical expression of unpaired electrons on molecules. Here, h is Planck’s constant, and its value is 6.626 × 10^−27^ erg/s; B is the universal constant, and its value is 9.274 × 10^−21^ erg/G; v is the frequency, and its value is 9.707 × 10^9^ Hz.

The ESR spectra recorded for the investigated sample indicated that the spectroscopic splitting factor (g-value) and the peak-to-peak width (ΔH_pp_) for the sample are found to be g = 2.969 and ΔH_pp_ ≅ 241 mT, respectively.

In the literature, low-spin and high-spin complexes for Fe^3+^ have been determined as 1.4–3.1 and 2.0–9.7, respectively [51]. It is seen that the g factor value of the synthesized mg-p(HEMA) nanoparticles is also compatible with the literature. According to the results obtained, it is understood that the synthesized nanoparticles have a local magnetic field in their structures [52].

#### 7.2.7. Zeta Size and Potential Analysis

Zeta size analysis of magnetic nanoparticles His-graft-mg-p(HEMA) developed with different strategies was analyzed with Nano Zetasizer (NanoS, Malvern Instruments, London, UK). For this purpose, particle zeta size measurement was performed by placing 1 mL of nanoparticle solution in the sample chamber of the zeta size analysis device [46].

**Figure 7:**
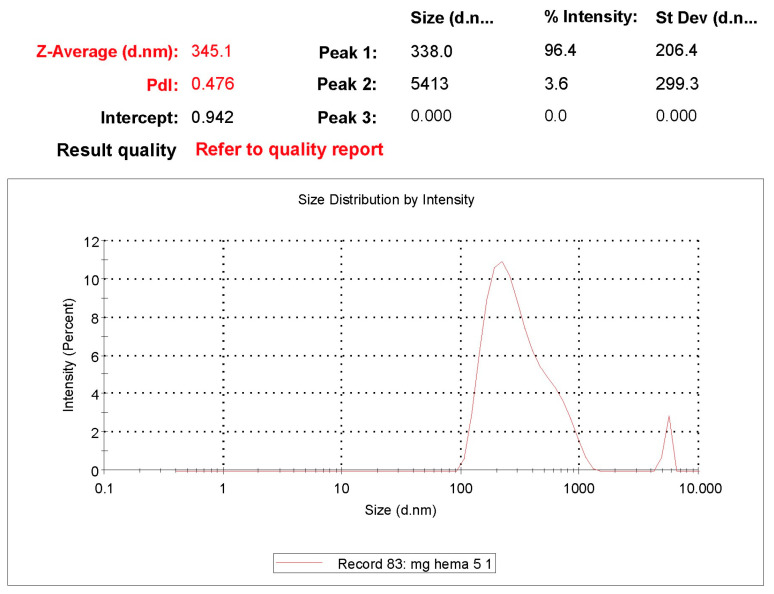

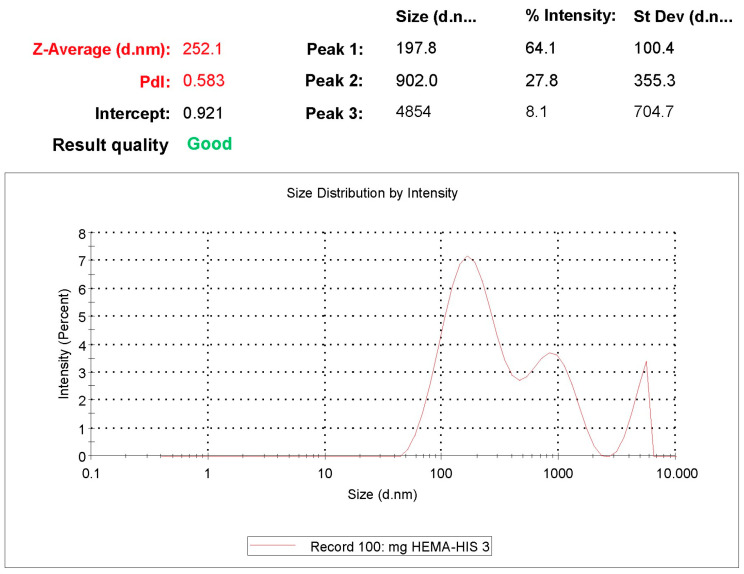
Zeta size analysis of mg-p(HEMA) and His-graft-mg-p(HEMA) magnetic nanoparticles.

As shown in Figure 7, the results obtained were large, consistent with the aggregation seen in SEM and AFM analyses. It is understood from this that the nanoparticles are dispersed as aggregates in liquid form as well. The most crucial factors in the process of aggregation are determined by the intensity of the external magnetic field. The proportional contribution of Stokes’ drag relative to the magnetophoretic force diminishes with increasing aggregation diameter, and the Brownian motion is almost nonexistent. This magnetically induced aggregation is required as it greatly improves the separation efficiency, in contrast to the aggregation that occurs during storage.

**Figure 8:**
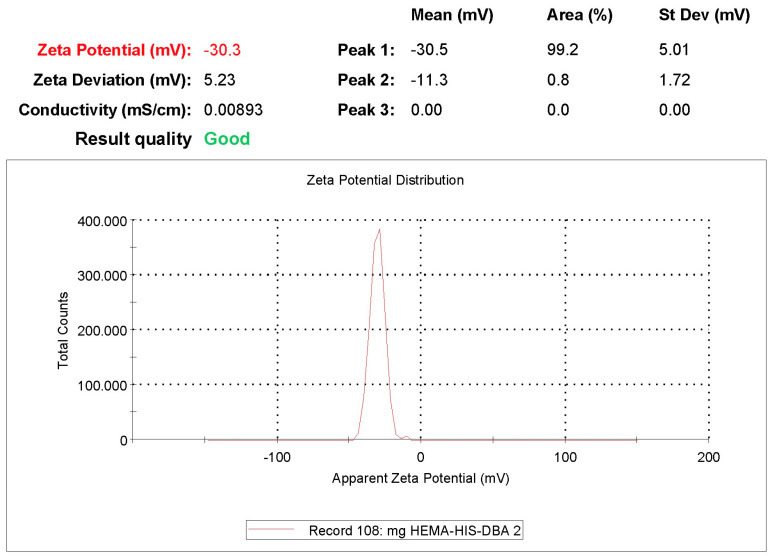
Zeta potential analysis of His-graft-mg-p(HEMA)-Cu^2+^-DBA magnetic nanoparticles.

Since there will be a change in angstrom levels (Cu^2+^ and DBA) in the ongoing modifications, size analysis was not required, considering that there would be no measurable significant difference in the size of the magnetic nanoparticles. Considering that the effects of these modifications (Cu^2+^, DBA) to be made by secondary interactions on magnetic nanoparticles will be electrostatic on the surface, these modifications were followed by zeta-potential analyses rather than zeta-size analyses (Figure 8).

Zeta-potential analyses were performed by suspending magnetic nanoparticles in ultrapure water (pH 8) and the findings are presented in the table.

As can be seen from Table 4, when the zeta potentials on the surface are examined before and after DBA bonding to the magnetic nanoparticle surface, it is seen that there are significant differences. It is understood that DBA modifications cause a change in surface charge and bonding occurs. Zeta potential measurements for well-scattered NPs are more than +30 mV or lower than 30 mV [53].

### 7.3. Sterilization

The sterilization of the magnetic nanoparticles was performed with ethyl alcohol, and the modifications of Cu^2+^ and DBA were completed in the sterile cabinet. There was no turbidity when the tubes containing His-graft-mg-p(HEMA)-Cu^2+^-DBA NP, and the negative control tubes containing no contaminants were compared with the positive control tubes with non-sterile PBS added. There was turbidity of the nanoparticles in the contamination medium after 7 days. While turbidity occurs in TG and TS media containing non-sterile PBS prepared as a positive control. Samples were taken from sterilely produced NPs.

### 7.4. Cell Culture

#### 7.4.1. Calibration Curves

The mean ± standard deviation (SD) of four replicates is given as bar graphs. For statistical evaluation of the significance levels of the data obtained from the applied test, GraphPad Prism program (version 6, GraphPad Software, Inc., San Diego, CA, USA) and IBM SPSS Statistics (version 20, IBM SPSS Software, IBM Corp., Armonk, NY, USA) were used. Statistical differences between the experimental groups (*n* = 4) were evaluated with the one-way ANOVA (analysis of variance) method/*t*-test for those with normal distribution, with the Kruskal–Wallis test/Mann–Whitney U test for those with normal distribution at a 99% confidence interval (*p* ≤ 0.01), and Pairwise relations between groups were determined via Tukey’s post hoc and Dunn’s multiple comparison methods, respectively. Calibration curves can be found in the Supplementary file as Figure S2.

#### 7.4.2. Cytotoxicity Analysis Results for HEL293 Cell Line ad SAOS-2 Cell Line

**Figure 9:**
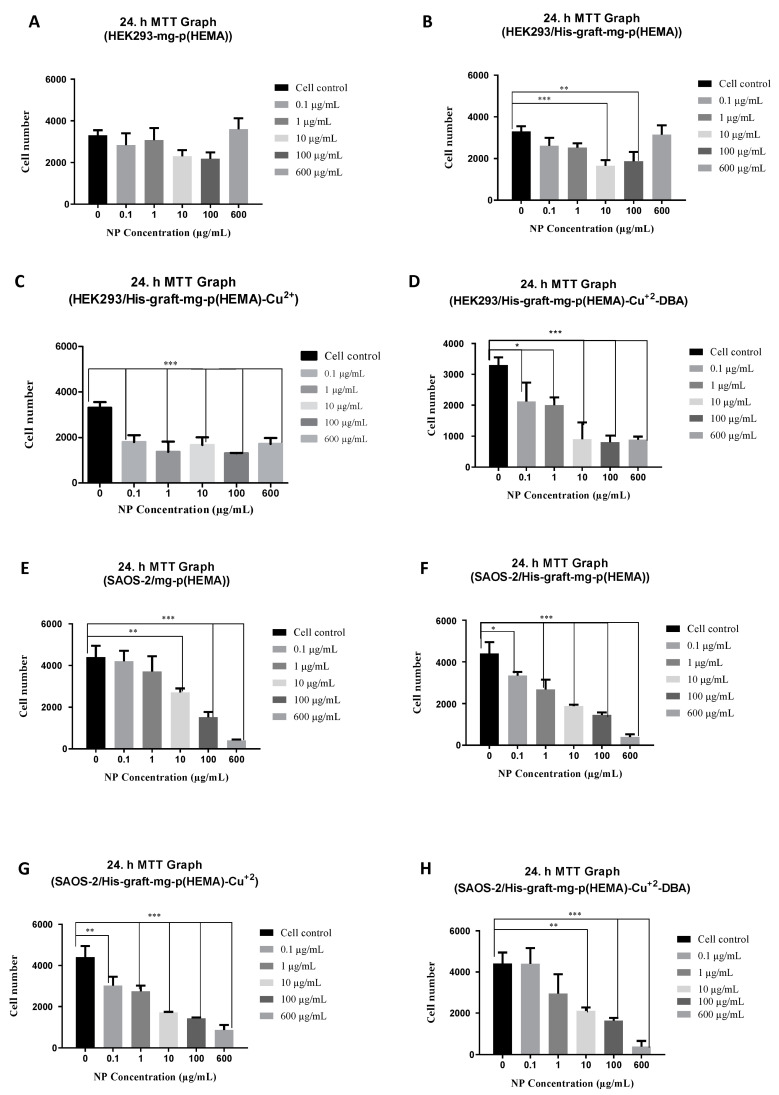
(**A**) A cell viability graph was obtained to examine the cytotoxic effect of mg-p(HEMA) nanoparticles on the HEK293 cell line. (**B**) A cell viability graph was obtained to examine the cytotoxic effect of His-graft-mg-p(HEMA) nanoparticles on the HEK293 cell line. (**C**) A cell viability graph was obtained to examine the cytotoxic effect of His-graft-mg-p(HEMA)-Cu^2+^ nanoparticles on the HEK293 cell line. (**D**) A cell viability graph was obtained to examine the cytotoxic effect of His-graft-mg-p(HEMA)-Cu^2+^-DBA nanoparticles on the HEK293 cell line. (**E**) A cell viability graph was obtained to examine the cytotoxic effect of mg-p(HEMA) nanoparticles on the SAOS-2 cell line. (**F**) A cell viability graph was obtained to examine the cytotoxic effect of His-graft-mg-p(HEMA) nanoparticles on the SAOS-2 cell line. (**G**) A cell viability graph was obtained to examine the cytotoxic effect of His-graft-mg-p(HEMA)-Cu^2+^ nanoparticles on the SAOS-2 cell line. (**H**) A cell viability graph was obtained to examine the cytotoxic effect of His-graft-mg-p(HEMA)-Cu^2+^-DBA nanoparticles on the SAOS-2 cell line. In subfigure (**B**), at the end of 24 h, toxic effects were observed at 10 and 100 µg/mL concentrations, while other concentrations did not cause a significant difference in viability compared to cell control. (** *p* ≤ 0.001, *** *p* ≤ 0.0001). In subfigure (**C**), after 24 h, all nanoparticle concentrations caused a significant decrease in viability compared to cell control. (*** *p* ≤ 0.0001). In subfigure (**D**), toxic effects were observed at all concentrations after 24 h. (* *p* ≤ 0.01, *** *p* ≤ 0.0001). In subfigure (**E**), at the end of 24 h, toxic effects were observed at high concentrations (10–600 µg/mL), while other concentrations did not cause a significant difference in viability compared to cell control. (** *p* ≤ 0.001, *** *p* ≤ 0.0001). In subfigure (**F**), at the end of 24 h, there was a significant decrease in viability compared to cell control at all nanoparticle concentrations. (* *p* ≤ 0.01, *** *p* ≤ 0.0001). In subfigure (**G**), after 24 h, there was a significant decrease in viability relative to cell control at all concentrations. (** *p* ≤ 0.001; *** *p* ≤ 0.0001). In subfigure (**H**), after 24 h, there was a significant decrease in viability relative to cell control at all concentrations except the low concentrations of 0.1 and 1 µg/mL (** *p* ≤ 0.001, *** *p* ≤ 0.0001).

There was no significant difference in viability at any concentration after 24 h (Figure 9A).

Considering the cytotoxicity results for both cell lines, there was a decrease in viability as the nanoparticle concentration increased. Similarly, in a study with silica nanoparticles, the authors showed that the cytotoxic effect on human lung cells was dose-dependent, with a decrease in viability as the concentration increased [54]. In another study, the authors stated that iron oxide nanoparticles coated with polyvinylpyrrolidone started to have a cytotoxic effect on SH-SY5Y cells at the end of the 48th hour, and cell viability decreased as the concentration increased [55]. In a study by Sahu et al., the authors conducted the study with zinc oxide and silicon dioxide nanoparticles; because of the viability analysis of human lung epithelial cells (L-132) and human monocytes (THP-1), they showed that nanoparticles had a concentration-dependent cytotoxic effect on cells, and a decrease in viability occurred at high concentrations [56].

Considering the cytotoxicity results, different results were obtained in SAOS and HEK293 cell lines. These differences are thought to be particularly related to cell size. In a study investigating the effects of cell size on the cells’ engulfment of nanoparticles in the membrane, it was found that fluorescent dye-labeled particles of human mesenchymal stem cells whose diameters (20, 40, 60, and 80 µm) can be controlled because of the application of polyvinyl alcohol to certain points via the UV photolithography method in a polystyrene-coated cell culture dish. It has been shown that the amount of membrane penetration decreases from the cell with the largest diameter to the cell with the smallest diameter [57].

Similarly, in our study, it was observed that the His-graft-mg-p(HEMA)-Cu^2+^-DBA nanoparticles produced in the HEK293 cell line, which is smaller in size than the SAOS cell line, have more toxic effects.

It has been stated that the regular arrangement of hydrophilic and hydrophobic ligands on the particle surface facilitates passive entry for gold nanoparticles and lipid particles, and when the ligands are arranged in strips, the nanoparticles can easily migrate across the membrane, whereas endocytosis occurs in random arrangement [58]. Accordingly, the cytotoxicity differences in our study may also be due to the arrangement and density of the hydrophilic ligands on the nanoparticles. The nanoparticle size determines the mechanism of nanoparticle uptake into the cell (endocytosis). Particles >200 nm are mostly taken into the cell via phagocytosis, while smaller nanoparticles are taken into the cell via the pinocytic pathway [59,60]. More details, according to reports: NPs up to 150 nm are primarily internalized via clathrin-mediated endocytosis (CME) or caveolin-mediated endocytosis (CVME), with a size limit of 200 nm, while NPs between 250 nm and 3 m have shown to have an optimal in vitro uptake via macropinocytosis and phagocytosis [61].

In some studies, it has been stated that small nanoparticles are more toxic, while in some studies, the cytotoxic effect of large nanoparticles is higher [62,63,64]. Due to their surface charge, nanoparticles can interact with serum proteins in the medium and coagulate [65]. Agglomeration occurs at high concentrations [66]. For this reason, different concentrations of the same nanoparticles showed quite different cytotoxic effects from each other. It is thought that this may be due to changes in nanoparticle sizes with agglomeration and changes in their uptake mechanisms. Therefore, a calculated IC50 value could not be determined because there was no gradual change in nanoparticle cytotoxicity depending on the concentration and the results were fluctuating.

The toxic effect of nanoparticles may vary between different cell types in terms of differences in cell physiology (e.g., epithelial or lymphoid), proliferation status (tumoral or resting cells), membrane characteristics, and phagocyte characteristics. For example, cancer cells are more resistant to nanoparticle toxicity due to their higher proliferation rate and metabolic activity than normal cells. Differences can be seen in the toxic effect of nanoparticles obtained with the same material. For this reason, the evaluation of nanoparticle cytotoxicity should be performed according to the target organ/cell [67,68]. In our study, the results of cytotoxicity were evaluated only according to the data of the SAOS-2 cell line, and the experiments were continued according to the results of these data.

In addition to cell type, the size, shape, and concentration of nanoparticles affect cytotoxicity. Additionally, the toxicity is affected by the presence or absence of a shell and active groups on the surface. These parameters cause differences in cases such as nanoparticle uptake, protein absorption, and accumulation in organelles. One of the reasons for these effects is the correlation between particle size and surface area. The increase in the number of molecules expressed on the surface with the increase in the surface area changes the size properties of the nanoparticles. Apart from that, the agglomeration of nanoparticles, which occurs easily due to the high surface energy, causes the nanoparticles to increase from their original size. In addition to agglomeration, uncontrolled adsorption of proteins can cause quite a change in cell-particle interaction. For this reason, preserving the original size, shape, and surface properties of the particles is very important in obtaining more reliable results [67,69].

DBA acts in a cell type-specific manner. As a result of cytotoxicity studies performed with cell lines, it is generally stated that it is biocompatible. However, it showed little toxicity on CCL-220 (human colon cancer cell) and especially CRL-1459 (non-malignant human colon cell) cell lines. Consistent with the result we obtained in our study, it was stated that non-cancer cells were significantly more affected by lectin than cancer cells [70]. An increase in toxic effect occurred because of the modification of mg-p(HEMA) with histidine.

Copper-induced toxicity and carcinogenesis were investigated in a study with human liver carcinoma cells (HepG2). When the MTT results are examined, it is seen that it induces dose-dependent toxicity [71]. In one study, the authors worked with mouse fibroblast cells (L929) to investigate the concentration-dependent toxicity of copper about the sustained release of copper (Cu) ions from Cu-containing intrauterine devices (CuIUD). As a result of MTT analysis, they stated that there was a decrease in viability as the concentration increased, and the LD50 value was 46 µg/mL [72].

In a study using the CellTiter-Glo luminescence cell viability test on normal lymphocyte and leukemia cell lines, it was shown that DBA did not cause a toxic effect [73]. Similarly, in our study, a decrease in the toxic effect was observed because of the modification of nanoparticles with DBA.

It is very difficult to control the size, shape, stability, and dispersibility of nanoparticles in the desired solution. The fact that magnetic iron oxide nanoparticles have high energy due to their high surface/volume ratio causes them to acquire a tendency to form aggregates to reduce this surface energy. Uncoated iron oxide nanoparticles have high chemical activity and are easily oxidized, which usually results in the nanoparticles losing their magnetic effect and dispersibility. For this reason, it has been stated that it is very important to develop strategies that provide suitable surface coating and other stability protection [74,75]. In our study, toxicity was reduced because of modifying the nanoparticle surfaces with DBA.

#### 7.4.3. Fluorescent Activated Cell Sorting (FACS) Results

**Figure 10:**
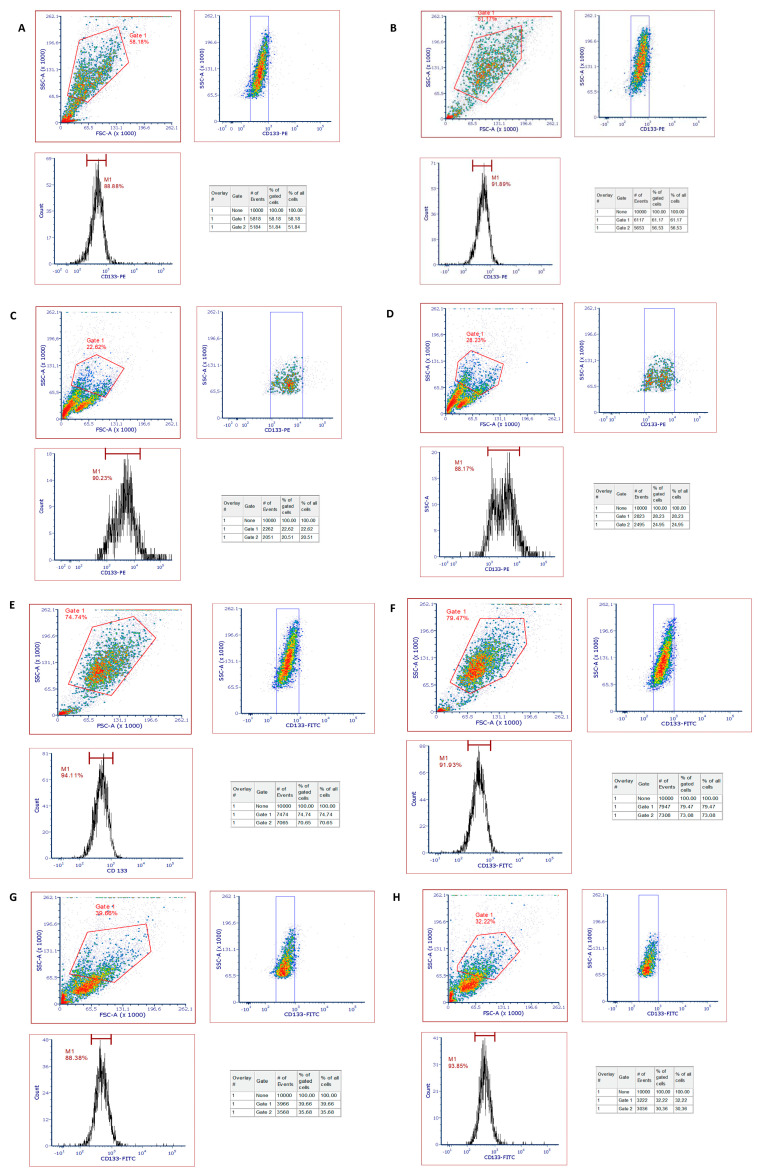
(**A**,**B**) Flow cytometry analysis of separation using commercial microbeads from 10^8^ cell counts—repetition 1 and 2. (**C**,**D**) Flow cytometry analysis of separation using commercial microbeads from 10^6^ cell counts—repetition 1 and 2. (**E**,**F**) Flow cytometry analysis of the separation was performed by using His-graft-mg-p(HEMA)-Cu^2+^-DBA nanoparticles at a concentration of 0.1 µg/mL from 10^8^ cell counts—repetition 1 and 2. (**G**,**H**) Flow cytometry analysis of the separation was performed by using His-graft-mg-p(HEMA)-Cu^2+^-DBA nanoparticles at a concentration of 0.1 µg/mL from 10^6^ cell counts—repetition 1 and 2.

**Figure 11:**
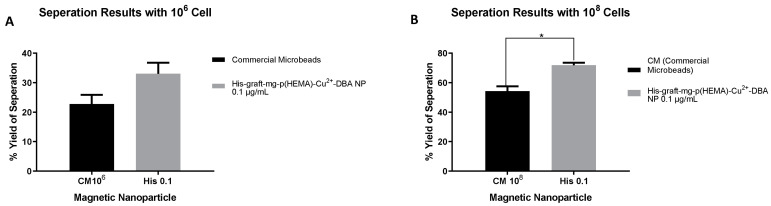
(**A**) % yield results of CD133^+^ cells separated from 10^6^ cells with commercial microbeads, 0.1 µg/mL His-graft-mg-p(HEMA)-Cu^2+^-DBA nanoparticles. (**B**) % yield results of CD133^+^ cells separated from 10^8^ cells with commercial microbeads, 0.1 µg/mL concentration of mg-p(HEMA)-HIS-Cu^2+^-DBA nanoparticles. (* *p* ≤ 0.05).

Considering the results of MACS-FACS analysis performed with 10^6^ and 10^8^ cell numbers (Figure 10), no statistically significant difference was observed in the CD133^+^ cell separation efficiency performed with nanoparticles and microbeads when the confidence interval was taken as 99%. However, when the confidence interval was taken as 95%, there was a statistically significant increase in the separation efficiency with His-graft-mg-p(HEMA)-Cu^2+^-DBA nanoparticles at a concentration of 0.1 µg/mL compared to commercial microbeads (Table 5 and Figure 11).

## 8. Discussion

Existing patents for similar purposes to our product were searched using “http://www.espacenet.com” (accessed on 23 June 2023) as the database and “http://www.patBASE.com” (accessed on 25 June 2023) as the patent information service. The research type was determined as “innovation research”, and the research title was named “High-efficiency, low-cost method in cancer stem cell isolation with magnetic functional nanoparticles”. During the patent search, “stem cell isolation with magnetic p(HEMA) nanoparticles, m-graft-his-poly (HEMA), lectin and Cu modification” were used as keywords. The patents that are thought to be close in terms of area after the examinations and the nanoparticles developed within the scope of the study are compared below.

In the patent numbered US2014134698A, “Method Of Extracting Neural Stem Cells Using Nanoparticles”, silica-coated magnetite Fe_3_O_4_ nanoparticles of approximately 100 nm were used for the separation of CD133^+^ neural stem cells. In the patent numbered US2018059114A “Magnetic Nanostructure For Detecting And Isolating Circulating Tumor Cells Comprising Antibody And Magnetic Nanoparticle Conjugated Conductive Polymer”, conductive polymers of antibodies and magnetic nanoparticles connected to the identification and isolation of circulating stem cells were used. Stem cell targeting and activation were carried out using magnetic nanoparticles in the patent numbered US2011034753A, named “Stem Cell Targeting And Activation Using Magnetic Particles”. “Miltenyi Biotec Gmbh” company, on the other hand, has patented the column design of the magnetic separation (MACS) system, and the magnetic nanoparticles they are entitled to are micro-sized beads with immunoaffinity properties.

In the concept of this study, we develop affinity-based nanoparticles for cancer stem cell isolation. For this purpose, a specific affinity recognition was used for the CD133, which is the stem cell biomarker. In addition to serving as a biomarker, CD133 also has roles in tumor biology, cell formation, and growth. Therefore, this developed system and strategy can be used not only the cancer stem cell separation; it also has the potential to be used in the characterization of the cancer stem cells and determining the CD133 levels. These applications would be meaningful in cancer research. In addition to the cancer stem cells, CD133 is also expressed in hematopoietic stem cells, endothelial progenitor cells, glioblastoma, neuronal and glial stem cells, pediatric brain tumors, adult kidney, mammary glands, trachea, salivary glands, uterus, placenta, digestive system, testes, and other cell types. So, this affinity-based recognition system is also useful for these cells’ separation and recognition.

Within the scope of the study, His-graft-mg-p(HEMA)-Cu^2+^-DBA nanoparticles synthesized and characterized for the isolation of stem cells were derivatized with different affinity ligands containing Cu^2+^ and DBA lectin. The yields of hand-held nanoparticles in stem cell isolation were compared. In this sense, nanoparticle design, synthesis, strategy, and modification have proven their originality by showing significant differences from the related patented products. In addition, our nanoparticles can recognize cells specifically, it has been observed that the related products have higher efficiency in stem cell isolation compared to the existing methods. Considering the cost in the nanoparticle synthesis and derivatization stages, it can be said that the products developed for this purpose will be low cost. In addition, the fact that the studies that can be closest in patent research were registered in 2014 shows the innovativeness of the study and the need in this field.

As a result of the study, the production of a magnetic nanoparticle with domestic production, advanced technology, and high efficiency compared to its current commercial competitor has been completed to reduce foreign dependency instead of imported microbeads. In this sense, the developed nanoparticles proved their originality by showing significant differences from the related patented products in terms of nanoparticle design, synthesis, strategy, and modification. In addition, because the products obtained within the scope of the project can recognize cells specifically, it has been observed that the related products have higher efficiency in stem cell isolation compared to the existing methods.

As a result, since the product obtained has the characteristics of recognizing cells specifically, it is expected that the stem cell isolation performed will be lower cost and higher efficiency compared to existing methods. Besides the fact that the products obtained in this study have the potential to obtain a patent, it is predicted that the stem cell isolation methods developed based on these products will be employed in the market as commercial products. This method, which was developed for the isolation of CSC, will be a low-cost and high-efficiency precursor for new cancer treatment methods to be developed.

## Figures and Tables

**Table 1:** ESR spectrometer operating conditions were adopted during the experiment.

Central Field	2700 G
Sweep width	5000 G
Microwave frequency	9.78 GHz
Microwave power	0.165 mW
Modulation frequency	100 kHz
Modulation amplitude	0.1 mT
Receiver gain	3.17 × 10^3^
Sweep time	83.89 s
Time constant	81.92 s
Conversion Time	163.84 ms
Temperature	Room Temperature

**Table 2:** AFM results in terms of Ra, Rq, and Rmax values.

Nanopolymer	Ra	Rq	Rmax
mg-p(HEMA)	0.01 µm	0.0153 µm	0.265 µm
His-graft-mg-p(HEMA)	0.0116 µm	0.0158 µm	0.216 µm

**Table 3:** EDS analysis of mg-p(HEMA) and His-graft-mg-p(HEMA).

mg-p(HEMA)			His-graft-mg-p(HEMA)		
Element	Weight %	Atomic %	Element	Weight %	Atomic %
**C**	48.99	61.35	C	18.06	33.45
**O**	37.15	34.92	O	2.36	3.75
**N**	NA	NA	N	31.35	43.59
**Fe**	13.86	3.73	Fe	48.23	19.21

**Table 4:** Zeta potential analyses of magnetic nanoparticles.

Nanoparticle	Zeta Potential (mV)
mg-p(HEMA)	−33.3
His-graft-mg-p(HEMA)	−30.5
His-graft-mg-p(HEMA)-Cu^2+^-DBA	−29.7

**Table 5:** MACS and FACS analysis results (for 10^6^ and 10^8^ cells).

Particle	Repetition Time	Concentration (µg/mL)	SaOS-2 Cell Number	Isolated CD133^+^ Cell Number	Separation Efficiency by Flow Cytometer (%)
Commercial microbeads	1	N/A	1 × 10^8^	3.5 × 10^7^	51.84
	2	N/A	1 × 10^8^	1.81 × 10^7^	56.53
His-graft-mg-p(HEMA)—Cu^2+^-DBA NP	1	0,1	1 × 10^8^	3.4 × 10^7^	70.65
	2	0,1	1 × 10^8^	2.5 × 10^7^	73.08
Commercial microbeads	1	N/A	1 × 10^6^	8.75 × 10^4^	20.51
	2	N/A	1 × 10^6^	2.5 × 10^4^	24.95
His-graft-mg-p(HEMA)—Cu^2+^-DBA NP	1	0,1	1 × 10^6^	8.75 × 10^4^	35.68
	2	0,1	1 × 10^6^	1.25 × 10^5^	30.36

## Data Availability

Data are contained within the article.

## Supplementary Materials

The following supporting information can be downloaded at https://www.mdpi.com/article/10.3390/polym16020196/s1, Figure S1: Dry mass graphs of magnetic nanoparticles: (a) mg-p(HEMA); (b) His-graft-mg-p(HEMA); Figure S2: Calibration Curves for HEK293 and SAOS-2 cell lines.
