# Supplementary material for: Affinity-Based Magnetic Nanoparticle Development for Cancer Stem Cell Isolation
Source: Polymers (Basel). 2024 Jan 9;16(2):196. doi: 10.3390/polym16020196 (PMC10818538; doi:10.3390/polym16020196)
Supplement: Supplementary file 1 [file polymers-16-00196-s001.zip › polymers-2609377-supplementary.pdf]

Supplementary File

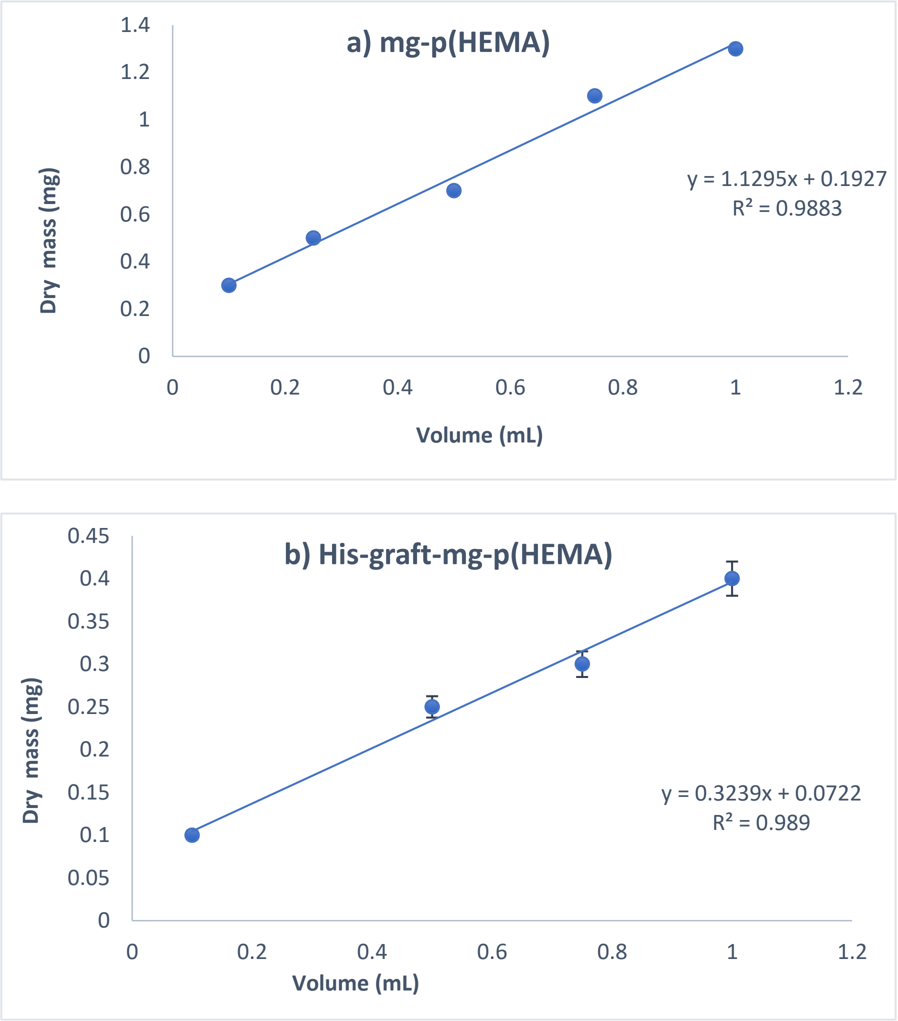

Figure S1. Dry mass graphs of magnetic nanoparticles a) mg-p(HEMA) b) His-graft-mg-p(HEMA).

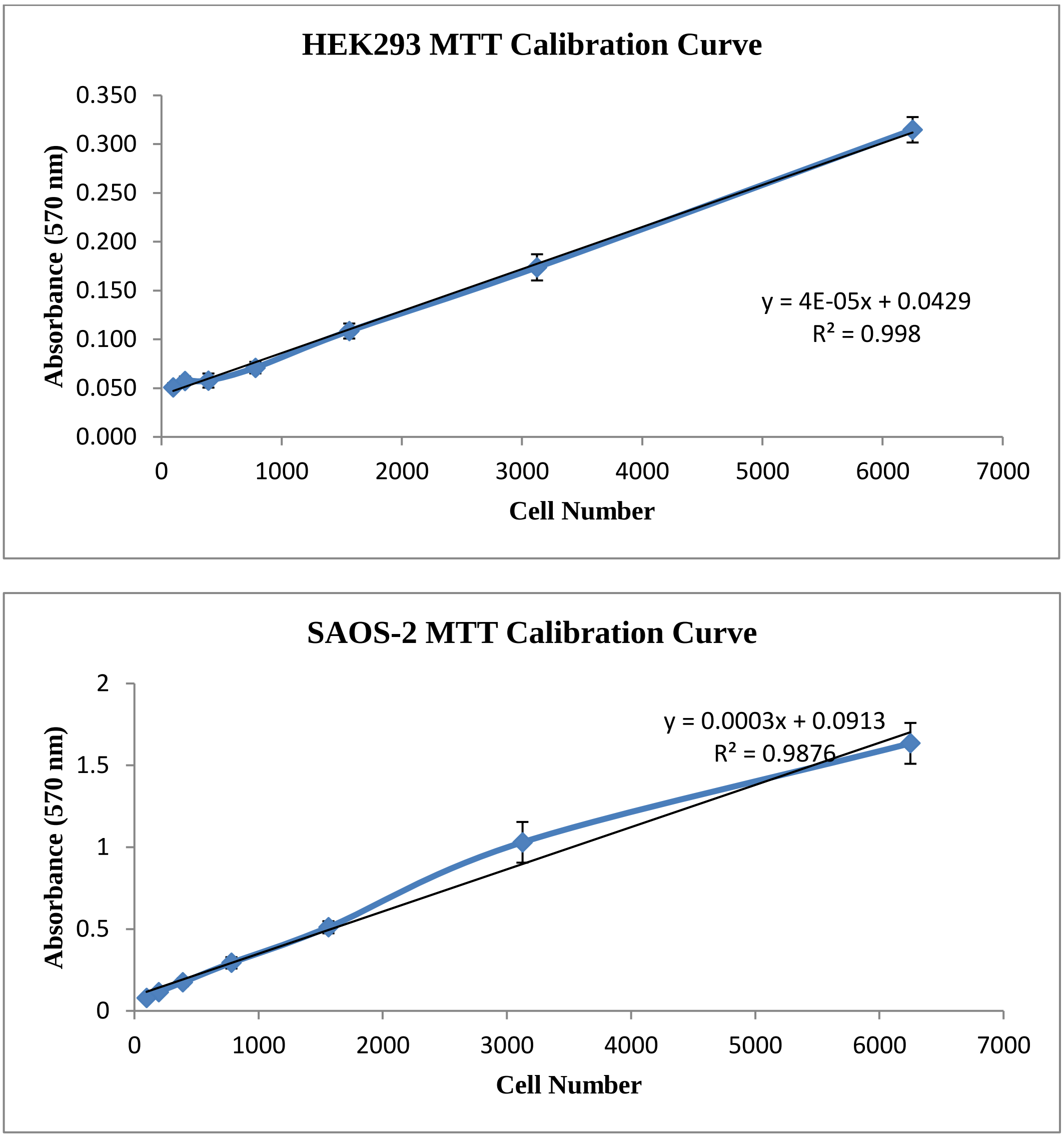

Figure S2. Calibration Curves for HEK293 and SAOS-2 cell lines.
